# Lung ultrasound and BNP to detect hidden pulmonary congestion in euvolemic hemodialysis patients: a single centre experience

**DOI:** 10.1186/s12882-020-02210-z

**Published:** 2021-01-21

**Authors:** Domenico Giannese, Alessandro Puntoni, Adamasco Cupisti, Riccardo Morganti, Enrico Varricchio, Claudia D’Alessandro, Claudia Mannucci, Piera Serio, Maria Francesca Egidi

**Affiliations:** 1 Nephrology, Transplant and Dialysis Unit, AOUP, Pisa, Italy; 2 Department of Clinical and Experimental Medicine, University of Pisa, Via Roma 67, 56126 Pisa, Italy; 3 Section of Statistics, AOUP, Pisa, Italy

**Keywords:** Pulmonary congestion, Hemodialysis, Lung ultrasound (LUS), Brain natriuretic peptide (BNP), Dry weight

## Abstract

**Background:**

Dry weight assessment in hemodialysis (HD) remains a challenge. The aim of the study was to investigate the prevalence of subclinical pulmonary congestion using lung ultrasound (LUS) in maintenance HD patients with no clinical or bioimpedance signs of hyperhydration. The correlation between B-lines Score (BLS) and brain natriuretic peptide (BNP) was also evaluated.

**Methods:**

Twenty-four HD patients underwent LUS and BNP dosage at the end of the mid-week HD session, monthly for 6 months . LUS was considered as positive when BLS was >15. Hospitalizations and cardiovascular events were also evaluated in relation to the BLS.

**Results:**

LUS+ patients at baseline were 16 (67%), whereas 11 (46%) showed LUS + in at least 50% of the measurements (rLUS+ patients). Only the rLUS+ patients had a higher number of cardiovascular events [*p*=0.019, OR: 7.4 (CI 95%. 1.32-39.8)] and hospitalizations [*p*=0.034, OR 5.5 (CI 95% 1.22- 24.89)]. A BNP level of 165 pg/ml was identified as cut-off value for predicting pulmonary congestion, defined by BLS >15.

**Conclusion:**

Prevalence of pulmonary congestion as assessed by LUS and persistent or recurrent BLS >15 were quite prevalent findings in euvolemic HD patients. In the patients defined as rLUS+, a higher rate of cardiovascular events and hospital admissions was registered. BNP serum levels > 165 pg/ml resulted predictive of pulmonary congestion at LUS. In the dialysis care, regular LUS examination should be reasonably included among the methods useful to detect subclinical lung congestion and to adjust patients’ dry weight.

## Background

The definition and adjustment of dry weight in hemodialysis (HD) patients is a real challenge for nephrologists. Evaluation of body water volume in HD patients is of relevant clinical significance. In fact, both hyper and hypo-hydration are linked with poor cardiovascular outcomes, especially if these conditions persist over time [1]. The estimation of body water is usually made by physical examination possibly coupled with body composition analysis by using bioelectrical impedance analysis (BIA). Physical examination includes the presence of dyspnea, shortness of breath, peripheral edema, rales at pulmonary auscultation, peritoneal or pleural effusion, or uncontrolled hypertension. Bioelectrical impendance analysis, both as multifrequence and as monofrequence technique, is able to estimate body fluid volumes and give clinician a valid instrument to asses changes in body fluids with time. Also BNP serum levels may be useful as marker of hyper-hydration; its concentration may be affected by multiple factors but it may be assumed as a marker of myocardial cell distension in response to circulating volume overload. Hence all these three methods may be affected by body volume changes, that is a crucial point in the real life care of the hemodialysis patients. While the use of BIA and BNP is of quite common use in dialysis practice, lung ultrasound (LUS) is an emerging innovative method that only recently has began to be used for the management of HD patients. LUS can detect extra vascular lung water, a relatively small but fundamental component of body fluid volumes and, at the same time, quantify lung congestion. This is the reason why LUS is playing an increasingly important role in the management of volume status in HD patients [2]. LUS can evaluate the presence of pulmonary microcirculation congestion, that is a frequent occurrence in HD patients, but often symptomless and not easily detectable [3, 4]. The increase in the pulmonary extravascular volume results in an air-water interface that induces an echo perceptible artefact known as B line [5]. The B line findings are of value for the diagnosis of pulmonary edema, already in its interstitial phase [6], and the detection allow to calculate a B line score (BLS) which is used to quantify pulmonary congestion [7]. There is a correlation between ultrafiltration volume and number of B lines at the end of the dialysis session. Noble et al. have demonstrated that the ultrafiltration induces a concomitant reduction of the B lines during the dialysis treatment [8]. This evidence shows that LUS is influenced by hydro electrolyte retention and body water. However, total water excess is not the only factor able to influence the formation of B lines. Also, cardiac parameters such as ejection fraction, atrial volume and pulmonary pressure are associated with B-lines detection [3]. In the HD population, LUS has predictive value for morbidity and mortality due to cardiovascular causes. It was found that sonography is more accurate than clinical score, as NHYA score, and it is also more accurate than BIA [9, 10]. Currently, a European multicenter trial is underway to test LUS, as systematic monitoring of “lung water”, to reduce mortality through correct definition of dry weight, also evaluating heart failure and acute cardiac injury, especially for high risk patients (history of myocardial infarction, stable and unstable angina, NYHA III-IV) (https://clinicaltrials.gov n.NCT02310061) [11].

The aim of this study was to evaluate the role of LUS in detecting the presence of subclinical pulmonary congestion in maintenance hemodialysis patients without clinical signs or bioimpedance indices of hyperhydration. Since LUS explore lung congestion, also the relationship between BLS at LUS and BNP serum level was examined.

## Methods

This was a prospective single-center cohort study including chronic HD patients on thrice weekly schedule. The study was performed from October 2018 to March 2019. The inclusion criteria were: age between 18 and 80 years old, clinical stability for at least 3 months, thrice-weekly hemodialysis treatment for at least 6 months, residual urine volume output less than 500 ml/day, clinical and bioelectrical impedance criteria of normohydration. Namely, patients were free of peripheral edema, dyspnea, rales at pulmonary auscultation, peritoneal or pleural effusion, uncontrolled hypertension, and with relative fluid overload (RFO) ranging from − 7% to + 7%. As exclusion criteria were assumed: cardiac devices or pacemakers, active infections, malignancy, metallic joint prostheses, chronic obstructive pulmonary disease or pulmonary fibrosis, history of thoracic surgery, left ventricular dysfunction with ejection fraction < 50%, upper or lower limb amputation, cognitive impairment or unwillingness to participate in the study. From the 111 patients treated in our HD center, 24 (21.6%) fulfilled the selection criteria and entered the study. Eighty seven patients were excluded for : pulmonary disease (13%), cardiac devices (6%), left ventricular function < 50% (10%), presence of malignancy (6%), absence of clinical stability (8%), major amputation (2%), clinical and instrumental hyperhydratation (15%), urinary output > 500 ml/day (2%), unwillingness to participate the study (15%), age > 80 years (13%) and other hemodialysis schedule (10%).

Dialysis vintage was reported as months from the time of dialysis commencing to the baseline of the study. The HD session was performed for 4 h, three times per week, using high-flux membrane dialyzers. The dialysate composition was sodium 140 mmol/L, potassium 2.5 mmol/L, calcium 1.5 mmol/L, bicarbonate 34 mmol/L and magnesium 0.5 mmol/L. All the patients underwent LUS examination and BNP dosage (at the end of the mid-week HD session), once a month, for 6 months. During this period, hospitalizations and cardiovascular events were also recorded and they included: myocardial infarction, acute heart failure, ECG-documented arrhythmias (defined according to the European Society of Cardiology), cerebrovascular accident or transient ischemic attack.

LUS were performed by a single investigator and during the follow-up period the patient's target weight was not influenced by the results of the examinations.

Bioelectrical impedance analysis was performed 30 minutes after the end of the HD session using a portable whole body bioimpedance spectroscopy device (BCM—Fresenius Medical Care D GmbH). Electrodes were placed on the side free from A-V fistula with the patient in supine position. The BCM measures the body resistance and reactance to electrical currents of 50 discrete frequencies, ranging between 5 and 1,000 kHz. Based on a fluid model using these resistances, the extracellular water (ECW), the intracellular water (ICW), and the total body water (TBW) are calculated. These volumes are then used to determine the amount of fluid overload. All calculations are automatically performed by the software of the BCM device. Absolute fluid overload (AFO) is the difference between the patient expected ECW under normal physiological conditions and the actual ECW, whereas the relative fluid overload (RFO) was defined as the AFO to ECW ratio. Considering RFO, the hydration status was defined as follows [12]: RFO < -7%, hypohydration; RFO between -7% and +7% normohydration; - RFO > + 7%, hyperhydration.

Lung ultrasound measurements were performed after the hemodialysis session with the available sonography equipment (MyLab 30, Esaote Biomedica, with 7.5 Mhz linear probe). Patients were in supine position during the examination. Twenty-eight different lung windows were scanned in the midaxillary, anterior axillary, midclavicular and parasternal spaces of the right and left hemithoraces, from the second to the fourth (on the left) and to the fifth (on the right) intercostal spaces, as previously described [13]. The B-line sign was defined as an echogenic artifact with a narrow origin on the pleural line, deepening to the inferior border of the screen and coherent with respiratory movements. The total number of B-lines (B-lines-score or BLS) was the sum of the artifacts recorded in the 28 sectors that were explored [6, 7]. Assuming a BLS cut-off value of 15 [6], LUS examinations were considered as negative for pulmonary congestion when BLS ≤ 15 (LUS-) and positive for pulmonary congestion when BLS > 15 (LUS+).

Patients who were LUS + at more than 50% of examination along the 6 month period of the study were classified as recurrent LUS+ (rLUS+).

All the reported biochemical parameters were determined using our lab routine methods. BNP was determined using enzyme-linked immunoassay method and venous blood samples were collected at the end of the HD session [14]. The study was approved by the Ethics committees of Pisa University Hospital. The study conformed to the Declaration of Helsinki and all participants provided written informed consent before enrollment.

### Statistical analysis

The results are reported as mean ± standard deviation or median (interquartile range) when appropriate. Comparison between-groups was performed using Student “t” test and Chi-square test for categorical variables. The risk of cardiovascular events was analyzed using the Kaplan-Meier method. Event-free survival analysis of demographic and clinical factors was performed using the univariate Cox model and hazard ratio (HR) with 95% confidence interval. A multiple linear regression analysis comparing demographic and clinical factors with BLS as dependent variable was performed and the data were expressed as coefficient beta, 95% confidence interval (CI), P and regression coefficient (RC).

Receiver operating characteristics curve (ROC) analysis was performed to identify the diagnostic usefulness of BNP dosage in predicting pulmonary congestion. The statistic program was PRISM Graphpad and SPSS version 3. A *p* value < 0.05 was assumed to indicate statistical significance.

## Results

Demographic, clinical and biochemical characteristics of the study population are reported in Table 1. Underling renal diseases were: diabetic nephropathy 4 (17%), glomerulonephritis 9 (37%), hypertensive nephropathy 3 (12.5%), autosomal-dominant polycystic kidney disease 1 (4%) and 7 (29%) other pathological conditions including renal graft failure, chronic pyelonephritis and unknown causes. Among the studied patients, 23 had an AV Fistula and only 1 had a central venous catheter. Eighteen patients were on standard bicarbonate dialysis treatment and 6 on online hemodiafiltration. At the baseline assessment, 16 (67%) patients resulted LUS+. Table 1 shows similar epidemiological, clinical and laboratory characteristics between patients who were LUS + or LUS- at baseline.

**Table 1:** Main demographic, clinical and laboratory data of the whole study population and of the LUS + and LUS- groups. Data are expressed as mean ± SD or median (IR) when appropriate

	Total n 24	LUS + n 16	LUS - n 8	*p* value
Age, years	60.5 ± 18.1	58.7 ± 20.2	64.1 ± 13.1	0.51
Dialysis vintage, months	26.7 ( 43.7)	48.0 (43.5)	26.0 (12.5)	0.089
Male – (Female), n	15 (9)	11 (5)	4 (4)	0.37
Systolic blood pressure-preHD, mmHg	133 ± 21	127 ± 17	135 ± 22	0.38
Diastolic blood pressure-preHD, mmHg	69.8 ± 14.1	71.5 ± 11.8	69.0 ± 15.2	0.68
Heart rate-preHD, bpm	66.1 ± 10.3	70.5 ± 6.1	64.3 ± 11.3	0.15
Systolic blood pressure-postHD, mmHg	132 ± 24	123 ± 11	138 ± 18	0.11
Diastolic blood pressure-postHD, mmHg	72.2 ± 12.9	68.5 ± 9.8	74.1 ± 11.4	0.33
Heart rate-postHD, bpm	68.2 ± 10.3	72.2 ± 12.1	66.5 ± 10.2	0.23
Diabetes, n (%)	6 (25)	2 (33)	4 (67)	0.15
Patients on antihypertensive drugs, n (%)	17 (71)	11 (65)	6 (35)	0.75
Cardiovascular comorbidities, n (%)	6 (25)	5 (83)	1 (17)	0.31
Ultrafiltration volume, L	2.4 ± 0.7	2.4 ± 0.8	2.4 ± 0.6	0.92
Ultrafiltration volume, % of body weight	3.3 ± 1.1	3.3 ± 1.1	3.1 ± 1.1	0.68
Relative fluid overload, %	-5.0 ± 10.5	-5.1 ± 11.4	-4.8 ± 9.5	0.95
Kt/V	1.4 ± 0.1	1.4 ± 0.1	1.4 ± 0.1	0.67
nPCR, g/kg/day	1.1 ± 0.1	1.1 ± 0.1	1.1 ± 0.1	0.76
Hemoglobin, g/dl	10.5 ± 1.2	10.4 ± 1.3	10.8 ± 1.0	0.51
Serum PTH, pg/ml	126 (200)	171 (157)	180 ( 305)	0,59
Serum phosphorus, mg/dl	5.3 ± 1.6	5.1 ± 1.4	5.8 ± 2.1	0.32
Serum cholesterol, mg/dl	155 ± 45	162 ± 40	151 ± 48	0.58
Serum albumin, g/dl	3.6 ± 0.8	3.7 ± 1.0	3.4 ± 0.4	0.41
C Reactive protein, mg/dl	0.17 (0.76)	0.05 (0.65)	0.20 ( 0.51)	0.111
Serum BNP, pg/ml	252 (292)	330 (280)	94 (66)	0.003

*LUS *negative for pulmonary congestion (B line Score ≤ 15), *LUS+ *positive for pulmonary congestion (B Line Score > 15), *BNP *Brain Natriuretic Peptide, *HD *hemodialysis, *nPCR *protein catabolic rate normalized by body weight

**Fig. 1:**
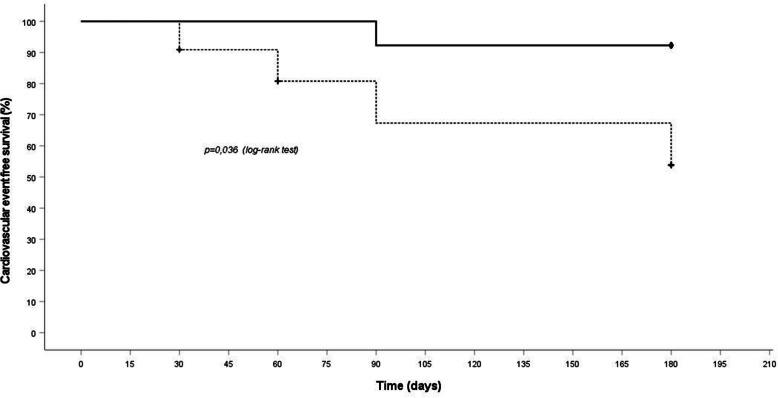
Days free from cardiovascular events in rLUS+ (dotted line) and not rLUS + (full line) patients. Patients were defined as rLUS + when B Line Score (BLS) > 15 in at least 50% of the measurements

**Fig. 2:**
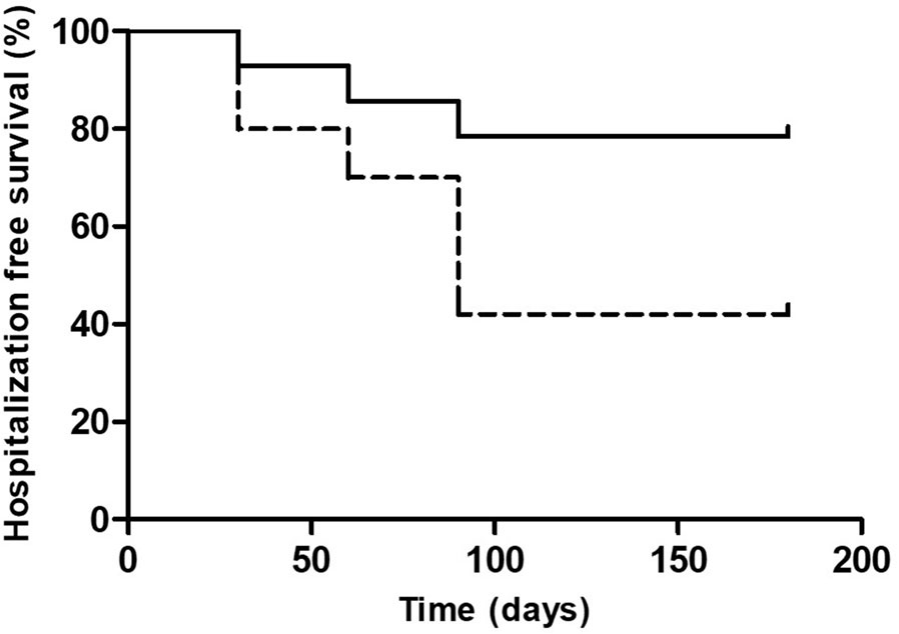
Days free from hospitalizations in rLUS+ (dotted line) compared to not LUS + (full line) patients. Patients were defined as rLUS + when B Line Score (BLS) > 15 was detected in at least 50% of the measurements

No statistically significant difference on hospitalization rate for any cause (*p* = 0.75) and for cardiovascular events (*p* = 0.12) was observed between LUS + and LUS– patients at baseline. Instead, only the patients with BLS > 15 in at least 50% of the measurements (11 patients) had a higher number of cardiovascular events [*p* = 0.019, OR: 7.4 (CI 95%. 1.32–39.8)] (Fig. 1) and of hospitalizations [*p* = 0.034, OR 5.5 (CI 95% 1.22–24.89)] (Fig. 2) along the study period.

Age, dialysis vintage, gender and prevalence of diabetes mellitus did not differ between rLUS + and not rLUS+ patients. Underlying cardiac comorbidities were not associated with the new-onset cardiac events (*p* = 0.13) or with the condition of rLUS+ (*p* = 0.24). Using the Univariate Cox model, gender, age, pre-existing heart disease, diabetes, dialysis vintage were not significantly associated with event-free survival, apart from hospitalization (*p* = 0.026) (Table 2).

**Table 2:** cardiovascular event free survival analysis of demographic and clinical factors

Factor	RC	*p*-value	HR (CI 95%)
Cohort (rLUS)	2	0,075	7,390 (0,816;66,9)
Age	-3,902	0,297	0,02 (0,001;31,1)
Gender	0,073	0,137	1,076 (0,977;1,184)
Pre-existing heart disease	1,073	0,242	2,925 (0,485;17,6)
Diabetes	1,662	0,07	5,269 (0,873;31,8)
Dialysis vintage	-0,005	0,7	0,995 (0,97;1.021)
Hospitalization	2,496	0,026	12,1 (1,3;109,7)

*RC *regression coefficient, *HR *hazard ratio, *95% CI *confidence interval

As expected, rLUS + group had a higher hospitalization rate than the remaining patients.

During the study, no patient died and 4 cardiovascular events were recorded. No association between cardiac diseases (such as hypertensive cardiopathy, ischemic heart disease, arrhythmias, miocardiopathy or others) and cardiac events (*p* = 0.13) was found. Furthermore we did not find any difference between LUS+ or LUS- patients considering cardiac pathology (*p* = 0.24). Cardiovascular events do not seem to be correlated with intrinsic cardiac pathology, with the limitation of the small sample size.

Considering all the measurements made, BNP serum levels resulted higher in the case of LUS+ (55 cases) than in LUS- (76 cases) findings. BNP resulted 122 ± 97 pg/ml (min 0, max 580 pg/ml) for LUS- patients and 547 ± 620 pg/ml (min 63, max 3500 pg/ml) for LUS+ patients. The distribution of the BNP values showed the following data: for LUS- patients the 25th percentile was 74.2 pg/ml, and the 75th percentile was 137 pg/ml (95% CI of mean: 99–144 pg/ml); for LUS+ patients the 25th percentile was 180 pg/ml, and the 75th percentile was 909 pg/ml (95% CI of mean: 379–714 pg/ml).

**Fig. 3:**
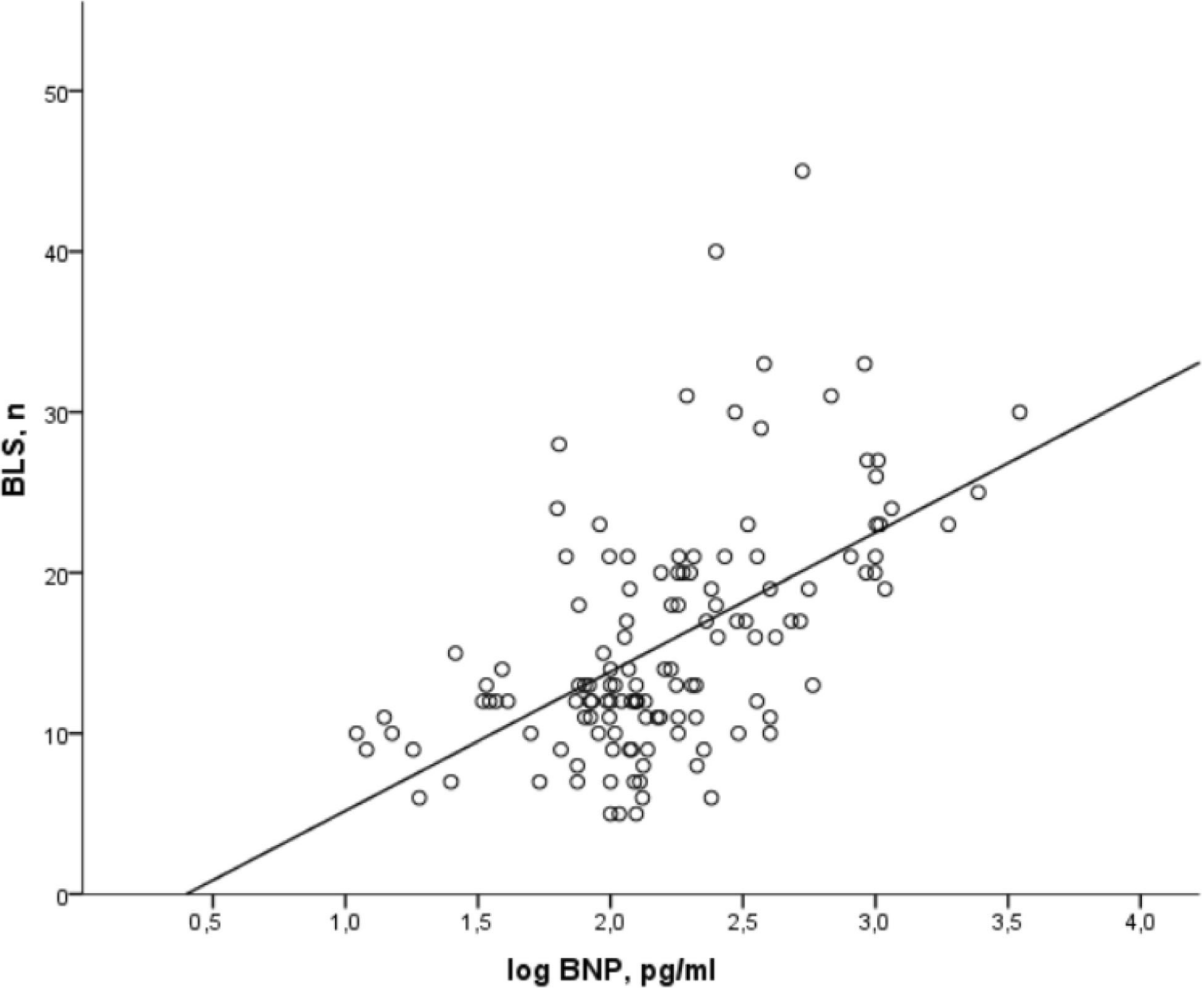
Correlation between B-lines Score (BLS) and Brain Natriuretic Peptide (BNP): *R* = 0,424, *R*^*2*^ = 0,18, regression coefficient = 6,809

Considering the total number of measurements (*n* = 131) performed in the patients during the whole follow-up, a positive correlation has been found between BNP and BLS (*r* = 0,424 *p* = 0.0001) (Fig. 3); since the BNP was not distributed normally, the values were analysed after log-transformation.

**Fig. 4:**
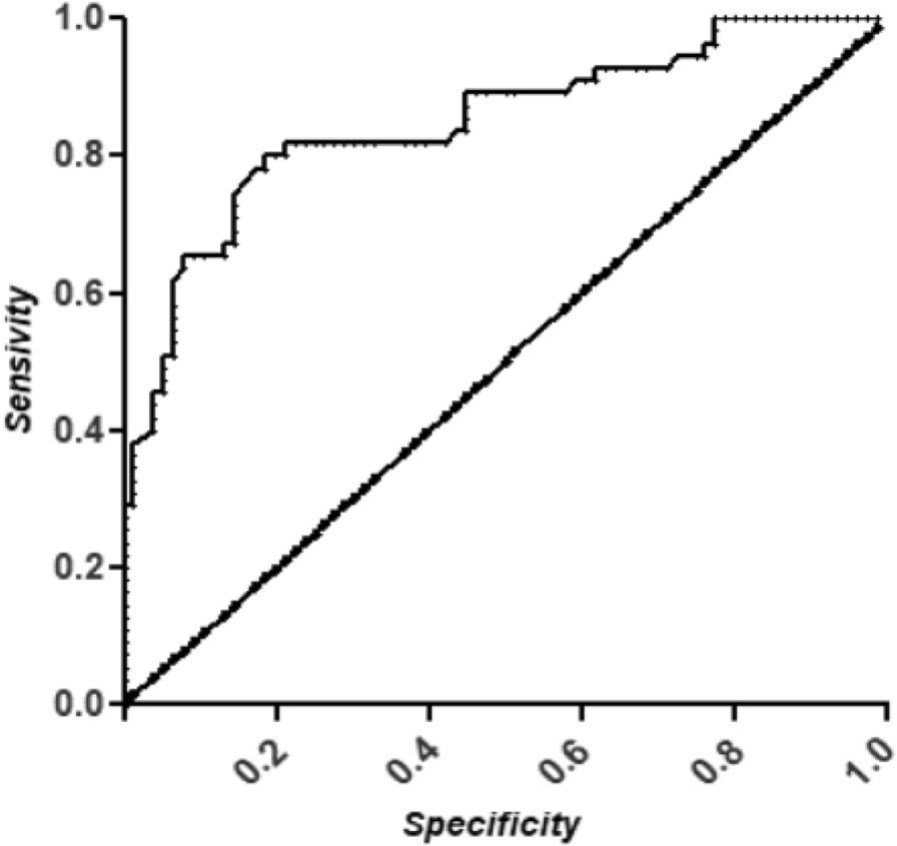
ROC curve of BNP serum level, in predicting pulmonary congestion

The ROC curve of BNP serum levels as predictor of pulmonary congestion is shown in Fig. 4. The area under the curve was 0.84 (confidence interval 0.77–0.91). The optimal BNP cut-off value for predicting pulmonary congestion resulted as 165 pg/ml (sensitivity 0.80 ,specificity 0.80, positive predictive value 0.74, negative predictive value 0.85 ).

## Discussion

This study shows that the presence of pulmonary congestion as detected by lung ultrasound is quite prevalent in HD patients, even when no clinical or instrumental signs of hyperhydration exist. In fact, 16 out of 24 patients, corresponding to 67%, presented a BLS > 15 at the end of the HD session, in the absence of edema, dyspnea or volume overload assessed by BIA. This result agrees with the data that emerged in other studies performed on both hemo and peritoneal dialysis patients, and underlines the role and usefulness of LUS for the early detection of pulmonary congestion at a preclinical stage [15, 16]. In addition, no impairment of left ventricular function at echocardiogram was present, since it was assumed as exclusion criteria for enrollment. This is an important point, since Mallamaci et al. reported a significant correlation between the number of B-lines and various indices of cardiac performance in HD patients. They found that extravascular lung water was associated with the most relevant echocardiographic parameters, such as the left atrial volume, the E/E’ ratio and especially the ejection fraction, which remained, even after the multivariate analysis, closely associated with the BLS, both before and after the HD session [3]. While in the cohort studied by Mallamaci et al. a significant percentage of patients showed peripheral edema (48%), dyspnea with NYHA ≥ 3 (25%), and volume overload assessed by BIA (33%), patients in our study had a preserved left ventricular systolic function (EF > 50%), an optimal volemic control assessed by BIA measurement (RPO between − 7 and + 7%) and no clinical signs of hyperhydration. As a whole, our findings suggest that a subclinical pulmonary congestion may be present in a number of HD patients that were clinically considered to be at target body weight. Other observational studies investigated the relationship between BLS and echocardiography parameters, and they lead to different conclusions. Siripol et al. found an association between the main echocardiographic indices (LVFE, E/E ‘and left atrial volume) and post-dialysis BLS [10]. In contrast, Saad et al. did not detect a significant association between the BLS, calculated at the end of a HD session when patients were at their clinically determined dry-weight, and the systolic and diastolic cardiac function, assessed respectively by LVFE and the E/E’ ratio [17]. Since B-lines may be expression of either systemic hyperhydration or cardiac dysfunction, the data which emerged from our study can probably be explained by a mismatch between the volemic status and cardiac performance, undetectable by bioimpedance analysis, but intercepted by lung ultrasound examination. Finally, the increased lung permeability which is typical of HD patients, could explain the high prevalence of hidden lung congestion [18]. In any case, the detection of BLS > 15 should lead to the reduction of the target dry weight. The persistence of BLS in at least 50% of the measurements was predictive of a greater number of cardiovascular events and hospitalizations. Thus, it seems that persistence or recurrence of LUS positivity is an unfavorable prognostic factor. Other AA have evaluated the usefulness of BLS as a prognostic marker. Zoccali et al. showed, in a multicenter study, that the degree of lung congestion was a better predictor of mortality, cardiovascular events and hospitalizations than NYHA score. They showed that patients with very severe lung congestion (pre-HD BLS > 60) had increased risk for both all-cause mortality and cardiac events, even after adjustments for clinical and biochemical parameters [9]. Siripol et al. confirmed those results, showing that a pre-HD BLS > 30 was the best predictor of mortality when compared to echocardiography parameters and hydration status assessed by BIA [10]. Also, considering BLS at the end of the HD session, other AA have observed an increased risk of mortality in patients with high BLS. This suggests that the removal of fluids and the prevention of pulmonary congestion are important strategies to improve the prognosis of HD patients [17, 19].

The systematic application of LUS in HD patients, even when cardiac function is not impaired, may represent a useful method for the monitoring of dialysis population in the clinical practice.

In addition, benefits for patients are represented by the safety of the method (in comparison with chest X ray or CT scan), the repeatability, the easy bed-side performance, and the clinical relevance of information that can give.

In the present study, BLS showed a positive relationship with BNP values. The relationship between BLS and BNP values was already assessed in two studies on HD patients but the results were conflicting. Donadio et al. observed a positive association between BNP and BLS only when BNP was determined post-HD session, and not with pre-HD BNP levels [20]. Instead, Basso et al. showed no significant relationship between BLS and BNP values both pre and post dialysis. In our study, a relationship between post-HD BNP and BLS was found [21].

In addition, a BNP of 165 pg/ml was identified as a cut-off value to distinguish patients with or without pulmonary congestion, defined as BLS > 15. This suggests that BNP levels may be a valid surrogate which is able to suggest the presence of pulmonary congestion when LUS in not available.

As clinical-practice algorithm applicable in HD patients, the first step is the research for signs of overhydration, as shortness of breath, lower limb edema or increase of arterial blood pressure and as second step is the body composition analysis by BIA. When they are negative for hyperhydration, the use of LUS is preferable to BNP dosage to detect signs of hyperhydration and lung congestion.

Unfortunately, a number of barriers exist preventing a widespread implementation of LUS procedure in daily clinical practice. Namely, the availability of sonography, the training and expertise of the operator, the need of a longer stay of the patients in the hospital. The chance of LUS examination at bedside and during or immediately after the end of the dialysis section is a possible way to increase its applicability. As a whole, the implementation of LUS assessment may be a useful a safe strategy for the fluid status monitoring of HD patients.

The novelty of our study consists in the repeated measurements of BLS at LUS, on a monthly basis, and BNP at the same times, and in the selection of patients in stable clinical conditions in the absence of physical and instrumental signs of hyperhydration or heart failure. The main limitations of this study are the small sample size that prevent from drawing solid conclusions, and the single-center design; a bias due to reverse causation may alter the predictive role of LUS + in respect to events.

## Conclusions

In summary, a number of patients who were clinically euvolemic and believed at target dry weight demonstrated lung congestion at LUS in the absence of edema, dyspnea, hyperhydration as assessed by BIA or impaired left ventricular function. This suggests that patients at their supposed target weight may still have a residual volume overload that can be intercepted by LUS assessment. This finding is also of relevance because LUS+ is associated with a poor prognosis, and when LUS is not available BNP dosage may be of some help. Thus, it would be important that LUS have a key role in the clinical practice and become part of the nephrologist’s knowledge, in order to provide additional information able to personalize ultrafiltration volume in HD patients. This is particularly true in patients who apparently do not seem to need a reduction in dry weight when using current clinical and instrumental approaches. This study was exploratory and therefore a small sample of patients was recruited. The data we detected in hemodialysis euvolemic patients need confirmations by further studies including more centers and larger number of patients.

## Data Availability

All data generated or analysed during this study are included in this published article.

## Abbreviations

BIA: Bioelectrical Impedance analysis
BLS: B-line score
BNP: Brain nutriuretic peptide
CKD: Chronic Kidney Disease
HD: Hemodialysis
LUS: Lung UltraSound
NYHA: New York Heart Association
